# Circadian Variation of Plasminogen-Activator-Inhibitor-1 Levels in Children with Meningococcal Sepsis

**DOI:** 10.1371/journal.pone.0167004

**Published:** 2016-11-28

**Authors:** Navin P. Boeddha, Gertjan J. Driessen, Marjon H. Cnossen, Jan A. Hazelzet, Marieke Emonts

**Affiliations:** 1 Intensive Care and Department of Pediatric Surgery, Erasmus MC-Sophia Children’s Hospital, University Medical Center Rotterdam, Rotterdam, the Netherlands; 2 Department of Pediatrics, Division of Pediatric Infectious Diseases & Immunology, Erasmus MC-Sophia Children’s Hospital, University Medical Center Rotterdam, Rotterdam, the Netherlands; 3 Department of Pediatrics, Division of Pediatric Hematology, Erasmus MC-Sophia Children’s Hospital, University Medical Center Rotterdam, Rotterdam, the Netherlands; 4 Department of Public Health, Erasmus MC, University Medical Center Rotterdam, Rotterdam, the Netherlands; 5 Paediatric Infectious Diseases and Immunology Department, Great North Children's Hospital, Newcastle upon Tyne Hospitals NHS Foundation Trust, Newcastle upon Tyne, United Kingdom; 6 Institute of Cellular Medicine, Newcastle University, Newcastle upon Tyne, United Kingdom; Institut d'Investigacions Biomediques de Barcelona, SPAIN

## Abstract

**Objective:**

To study whether the circadian variation of plasminogen-activator-inhibitor-1 (PAI-1) levels, with high morning levels, is associated with poor outcome of children with meningococcal sepsis presenting in the morning hours.

**Design:**

Retrospective analysis of prospectively collected clinical and laboratory data.

**Setting:**

Single center study at Erasmus MC-Sophia Children’s Hospital, Rotterdam, the Netherlands.

**Subjects:**

184 patients aged 3 weeks to 18 years with meningococcal sepsis. In 36 of these children, PAI-1 levels at admission to the PICU were measured in plasma by ELISA.

**Interventions:**

None.

**Measurements and main results:**

Circadian variation was studied by dividing one day in blocks of 6 hours. Patients admitted between 6:00 am and 12:00 am had increased illness severity scores and higher PAI-1 levels (n = 9, median 6912 ng/mL, IQR 5808–15600) compared to patients admitted at night (*P* = 0.019, n = 9, median 3546 ng/mL, IQR 1668–6118) or in the afternoon (*P* = 0.007, n = 7, median 4224 ng/mL, IQR 1804–5790). In 184 patients, analysis of circadian variation in relation to outcome showed more deaths, amputations and need for skin grafts in patients admitted to the PICU between 6:00 am and 12:00 am than patients admitted during the rest of the day (*P* = 0.009).

**Conclusions:**

Circadian variation of PAI-1 levels is present in children with meningococcal sepsis and is associated with illness severity, with a peak level in the morning. Whether circadian variation is an independent risk factor for morbidity and mortality in meningococcal sepsis needs to be explored in future studies.

## Introduction

Meningococcal endotoxins and the subsequent inflammatory host response induce excessive coagulation and downregulation of fibrinolysis in meningococcal sepsis. Hence, the delicate balance between coagulation and anti-coagulation shifts towards thrombosis and widespread deposition of fibrin throughout the microcirculation, with thromboembolism contributing to the need to amputate extremities, multiple organ dysfunction, and eventually death. [1]

In meningococcal sepsis, plasminogen-activator-inhibitor-1 (PAI-1) levels are increased and result in inhibited fibrinolysis and impaired anticoagulant mechanism, since PAI-1 neutralizes activated protein C [2], leading to severe disseminated intravascular coagulation (DIC). [3, 4] The *PAI1* 4G/5G polymorphism is associated with PAI-1 levels and with outcome. The highest risk for mortality is present in 4G/4G homozygous individuals, who produce the highest levels of PAI-1. [3]

The genotype is one of the multiple mechanisms which influences PAI-1 levels. Physiologically, levels are also subject to circadian variation, causing a PAI-1 peak in the morning. [5, 6] In meningococcal sepsis, clustering of fatal cases in the morning hours has been reported repeatedly. These findings are generally interpreted as the result of delayed detection of symptoms and subsequent health care seeking behavior, as signs of severe illness may easily be overlooked in the night or early morning hours. [7, 8] We hypothesize that the circadian variation of PAI-1, with high morning levels, is associated with excess mortality of cases presenting in the morning hours, possibly due to aggravation of multiple organ failure secondary to more severe DIC.

Here, we aim to study the circadian variation of PAI-1 levels in children with meningococcal sepsis in relation to outcome.

## Materials and Methods

We retrospectively analyzed clinical and laboratory data of a cohort of 184 patients aged 3 weeks to 18 years with meningococcal sepsis, who were enrolled in Rotterdam based meningococcal studies from 1988 to 2005. [3, 9] All patients fulfilled internationally agreed criteria for sepsis. [10] Meningococcal sepsis was diagnosed clinically (n = 28) and/or by positive culture or PCR from sterile sites (n = 156). In 36 of these children, PAI-1 levels at admission to the PICU were measured in plasma by ELISA as described before. [3, 9] Blood samples were taken on admission to the PICU, processed on ice and stored at -80 degree Celsius until analysis. [11] All studies were approved by the ethical committee of Erasmus MC, and written informed consent was obtained from parents or legal guardians.

Circadian variation was studied by dividing one day in blocks of 6 hours. Illness severity was indicated by the probability of death based on the BEP score (P (death BEP)) [12], DIC score [13], and Pediatric Risk of Mortality (PRISM) [14]. Quantitative variables are presented either as mean (±SD) when normally distributed or as median (IQR). For normally distributed variables, t-tests were used to compare two groups, while for non-normal variables the Mann-Whitney U test was used. To compare PAI-1 levels and illness severity between four time periods, we used a One-Way ANOVA when the depending variable was normally distributed and the Kruskal-Wallis test when the depending variable was non-normally distributed. The correlation between PAI-1 and illness severity was studied by Pearson’s (for normally distributed variables) or Spearman’s (for non-normally distributed variables) correlation. Chi squared tests or Fisher’s exact tests-in case of small sample size-were used to assess the association between two categorical variables. Data were analyzed using SPSS version 21.

## Results

Baseline characteristics of the total cohort of 184 patients and the 36 children in whom PAI-1 levels were measured (PAI-1 cohort) are presented in Table 1. Both groups had similar demographics, but illness severity in the PAI-1 cohort was higher as reflected by higher P (death BEP) and higher DIC score at admission.

**Table 1:** Baseline characteristics of all patients (n = 184) and the PAI-1 cohort (n = 36).

	All patients (n = 184)	PAI-1 cohort (n = 36)	*P*
**Age (median, IQR)**	3y (18m-8y)	2y (12m-9y)	ns
**Sex (% male)**	59%	56%	ns
**P (death BEP) (median, IQR)**	0.05 (0.03–0.12)	0.09 (0.04–0.23)	<0.01
**DIC score at admission (mean, ±SD)**	4.7 (1.9)	5.6 (2.0)	<0.05

Abbreviations: P (death BEP) = Probability of death based on the BEP score, DIC = Disseminated intravascular coagulation, IQR = Interquartile range, SD = Standard deviation, m = month(s), y = year(s).

**Fig 1:**
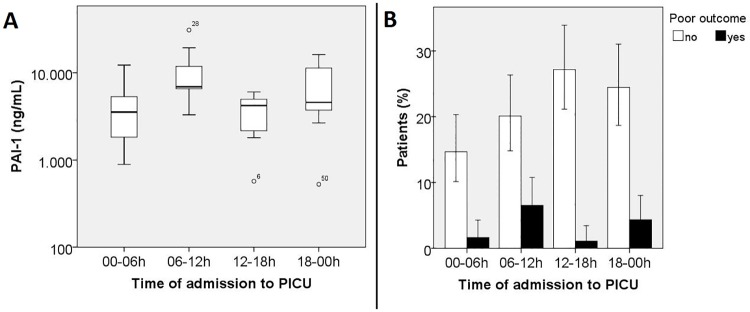
Time of admission to PICU in relation to plasminogen-activator-inhibitor-1 levels and outcome. (A) Patients admitted between 6:00 am and 12:00 am had higher plasminogen-activator-inhibitor-1 levels than patients admitted at night (*P* = 0.019) or in the afternoon (*P* = 0.007). (PAI-1 level on y-axis in logarithmic scale. Bar within box represents median, box represents Q1-Q3, whiskers represent 1.5*IQR, dots are outliers) (B) Outcome in patients admitted to the PICU between 6:00 am and 12:00 am was worse than patients admitted during the rest of the day (*P* = 0.009). (Error bars represent 95% CI)

Patients admitted between 6:00 am and 12:00 am had higher PAI-1 levels (n = 9, median 6912 ng/mL, IQR 5808–15600) than patients admitted at night (*P* = 0.019, n = 9, median 3546 ng/mL, IQR 1668–6118) or in the afternoon (*P* = 0.007, n = 7, median 4224 ng/mL, IQR 1804–5790). (Fig 1A) The distribution of *PAI1* 4G/5G genotype (*P* = 0.71) and allele frequency (*P* = 0.72) did not differ between four time periods.

In concordance with the variation in PAI-1 levels, patients admitted between 6:00 am and 12:00 am had an increased illness severity based on the BEP score (median P (death BEP) 0.26, IQR 0.16–0.44) than patients admitted at night (*P* = 0.009, median P (death BEP) 0.05, IQR 0.02–0.15), patients admitted in the afternoon (*P* = 0.001, median P (death BEP) 0.05, IQR 0.04–0.05), or patients admitted in the evening (*P* = 0.037, median P (death BEP) 0.10, IQR 0.04–0.24). Illness severity reflected by the DIC score did not differ between four time periods (*P* = 0.13). Moreover, illness severity correlated to PAI-1 levels (correlation between BEP score and PAI-1: r = 0.77, n = 36, *P*<0.001; correlation between DIC score and PAI-1: r = 0.67, n = 34, *P*<0.001).

The morning PAI-1 peak level and the morning peak in illness severity in 36 children was not associated with poor outcome, as defined by deaths, amputations and/or need for skin grafts (*P = 0*.24). To increase power, we analyzed circadian variation in the total group of 184 patients, and found a worse outcome in patients admitted to the PICU between 6:00 am and 12:00 am than patients admitted during the rest of the day (*P* = 0.009). Of the 184 patients, 49 patients were admitted between 6:00 am and 12:00 am, of whom 12 patients (7%) eventually had a poor outcome (6 deaths, 4 amputations and 2 skin grafts). This in contrast to 135 patients who were admitted during the rest of the day, of whom 13 patients had a poor outcome (00-06h: 2 deaths and 1 amputation (2%); 12-18h: 2 amputations (1%); 18-00h: 4 deaths, 3 amputations and 1 skin graft (4%)). (Fig 1B) Patients admitted between 6:00 am and 12:00 am showed a trend for higher PRISM, BEP score, and DIC score compared to patients admitted during the rest of the day. (PRISM 21.3 (±11.0) vs 16.1 (±10.0), *P* = 0.06; P (death BEP) 0.06 (IQR 0.02–0.20) vs 0.04 (IQR 0.03–0.10), *P* = 0.16; DIC 5.2 (±1.6) vs 4.5 (±2.1), *P* = 0.09)

Data underlying the findings from this study can be found in S1 File.

## Discussion

This study provides insight into the circadian variation of PAI-1 levels in children with meningococcal sepsis, and shows a significant PAI-1 peak level in the morning. Because the distribution of *PAI1* 4G/5G genotype and allele frequency, known to be associated with PAI-1 levels [3], did not differ between time periods, it is likely that PAI-1 levels in meningococcal sepsis are associated with circadian variation. However, numbers are limited and we cannot exclude other factors also influencing PAI-1 levels in these patients. [15]

Illness severity is one of the main factors associated with PAI-1 levels and multiple studies have associated high PAI-1 levels with increased illness severity. [3, 16–19] Also in our cohort, we found a strong correlation between PAI-1 levels and illness severity. Thus, given the correlation between PAI-1 and illness severity, PAI-1 could either be a marker for illness severity or a contributor to illness severity. [20] Future studies should specify the role of circadian variation of PAI-1 levels in meningococcal sepsis severity.

Our results show that patients admitted to the PICU in the morning have a worse outcome than patients admitted during the rest of the day. These results of outcome are in line with a retrospective epidemiological study from Western Norway, where meningococcal sepsis patients, both adults and children, hospitalized between 7:00 am and 11:00 am had a poorer prognosis than those admitted during other hours of the day. [8] Although multiple factors could have influenced increased morning severity, especially a possible delay in detection of symptoms during the night and early morning, in our opinion, morning PAI-1 peak levels might have contributed to this effect.

Scheer and Shea [5] reported a true endogenous circadian rhythm in circulating PAI-1 independent of behavioral and environmental factors. Absolute values of healthy adults had a peak-to-trough amplitude of 1.24 ng/mL, corresponding with an increase from trough to peak of 124%. In our cohort, the lowest median PAI-1 value of a time period was 3546 ng/mL, which increased to a peak value of 6912 ng/mL in the morning, corresponding with an increase of 95%. The extremely high PAI-1 level in meningococcal sepsis compared to patients with meningitis alone or healthy controls has been described before. [3] However, this is the first report describing that circadian variation of PAI-1 levels—and associated circadian variation of illness severity—is also present in meningococcal sepsis patients with extremely high PAI-1 levels.

In conclusion, our data demonstrate an association between circadian variation of PAI-1 levels and illness severity in pediatric meningococcal sepsis patients, with a significant peak level in the morning. Future study in a larger cohort of patients should focus on the question whether the morning PAI-1 peak is an independent risk factor for morbidity and mortality in meningococcal sepsis.

## Supporting Information

S1 File Data underlying the findings from this study. (SAV)
